# Severe anaemia and paediatric mortality after hospital discharge in Africa

**DOI:** 10.1016/S2352-4642(22)00103-1

**Published:** 2022-05-21

**Authors:** Moses M Ngari, James A Berkley

**Affiliations:** The KEMRI-Wellcome Trust Research Programme, Kilifi, Kenya; The Childhood Acute Illness and Nutrition Network, Nairobi, Kenya; The KEMRI-Wellcome Trust Research Programme, Kilifi, Kenya; The Childhood Acute Illness and Nutrition Network, Nairobi, Kenya; The Centre for Tropical Medicine and Global Health, Nuffield Department of Medicine, University of Oxford, Oxford OX3 7LG, UK

There is growing evidence suggesting that mortality after hospital discharge among children in low-income and middle-income countries (LMICs) is a major problem.^1–3^ Malaria and severe anaemia are leading causes of hospital admission and death in many parts of Africa with longer-term effects beyond the inpatient period.^1,2^ A previous systematic review of paediatric mortality after discharge in Africa in 2018 identified six papers reporting children admitted with severe anaemia, malaria, or both, but did not compare risks with other health conditions or perform a meta-analysis.^1^

In *The Lancet Child & Adolescent Health*, Titus K Kwambai and colleagues^4^ help to fill this gap with a systematic review and meta-analysis focused on children admitted to hospital in malaria-endemic settings in Africa with severe anaemia and other common conditions. Data from 20 of the 27 eligible studies suggested that all-cause mortality after discharge within 6 months occurred in 0·7% to 27·1% of children across a range of conditions. Remarkably, the range within conditions was almost as wide, with 2·3% to 18·8% for severe anaemia and 2·8% to 27·1% for severe malnutrition. HIV, hypoxia, bacteraemia, and leaving hospital against medical advice were identified as risk factors of mortality after discharge within the conditions examined. This review is a valuable contribution to our growing understanding of mortality and morbidity following hospital discharge and highlights the background of vulnerability among children before admission and after discharge from hospital. The concept of hospital admissions occurring within a child’s overall health trajectory^3^ is particularly well illustrated by the finding that showed that there is a 2·69-times higher risk of 6-month mortality after discharge associated with a previous admission for severe anaemia compared with admission for other health conditions.^4^

There was substantial variation in designs of the included studies; randomised controlled trials and cohorts with differing inclusion and exclusion criteria, clinical definitions (ie, severe anaemia was defined as haemoglobin <5 g/dL or <6 g/dL and malnutrition was defined using different criteria), and duration of follow-up. This reflects the timescale of publications since the 1980s with definitions for some conditions progressively changing. Publication bias could have occurred, but the authors were not able to assess this bias because of the few studies included. Likewise, the authors were not able to report on the causes of severe anaemia, the reasons for hospital readmissions, or causes of deaths after discharge. Data were also scarce on potential key predictors of mortality after discharge, such as socioeconomic status, HIV, other underlying conditions, caregiver health status, and other social and care-access challenges.

Mortality after discharge is well recognised among older populations and populations released from intensive care units in high-income settings as a transient vulnerability following hosptial admission, often referred to as post-hospital syndrome.^5^ Risks also vary by conditions and individual factors in those populations. It was notable that more than two-thirds of deaths occurred at home without a hospital readmission, posing a challenge in understanding the causes and reasons for not seeking medical care.^1,3^ These issues show the complexity of post-hospital syndrome. We consider that death after discharge may be caused by a new episode of illness, separate and unrelated to the index admission event, but reflecting ongoing susceptibility; incomplete or failed treatment of the reason for the index admission; or a condition that is acquired or exacerbated in hospital, such as a new infection, antimicrobial resistance, drug toxicity, or malnutrition, which later results in death (appendix). Because of the paucity of published data, Kwambai and colleagues^4^ were not able to disentangle these potential scenarios.

Some chronic diseases such as tuberculous, HIV, or severe malnutrition already have guidelines for follow-up. However, clinics focused on one condition commonly do not address broader health issues and biological or social vulnerabilities, and there are usually no defined follow-up guidelines for most children without these conditions.

Overall, this review, along with other papers on this topic, points to a need for better understanding of the epidemiology and mechanisms leading to deaths after discharge, and robust clinical trials testing the efficacy of interventions after discharge. The CHAIN Network recently showed that among acute paediatric admissions in general across Africa and south Asia, almost half of all deaths occur after discharge and that nutritional and social factors have important roles.^3^ Several trials addressing mortality after discharge in severe anaemia,^6^ severe malnutrition,^7^ and among all children being discharged^8^ have not shown benefits of micronutrient and antimicrobial interventions. However, two trials targeting severe anaemia and malaria in Malawi, Uganda, and Kenya have shown the protective effects of artemether-lumefantrine^9^ or dihydroartemisinin-piperaquine^10^ against all-cause mortality after discharge at 3 months. For adoption in guidelines, implementation and effectiveness trials of biological, psychosocial, and socioeconomic interventions targeting children at high risk of mortality after discharge and their families, including cost-benefit assessment, are needed.

## Supplementary Material

Supplementary Appendix
