# Supplementary material for: Severe anaemia and paediatric mortality after hospital discharge in Africa
Source: Lancet Child Adolesc Health. Author manuscript; Available in PMC 2022 Sep 21. (PMC7613614; doi:10.1016/S2352-4642(22)00103-1)
Supplement: Supplementary Appendix [file EMS153577-supplement-Supplementary_Appendix.pdf]

THE LANCET
Child & Adolescent Health

Supplementary appendix

This appendix formed part of the original submission. We post it as supplied by the authors.

Supplement to: Ngari MM, Berkley JA. Severe anaemia and paediatric mortality after hospital discharge in Africa. *Lancet Child Adolesc Health* 2022; published online May 20. https://doi.org/10.1016/S2352-4642(22)00103-1.

Appendix to: Severe anaemia and paediatric post-discharge mortality in Africa.

Post-discharge mortality usually occurs on a background vulnerability which may be medical, nutritional, social, or economic, including maternal physical or mental health challenges and a lack of access to health services.[1]

We consider that death after discharge may be due to i) a new episode of illness, separate and unrelated to the index admission event, but reflecting ongoing susceptibility; iii) incomplete or failed treatment of the reason for the index admission; or iii) a condition that is acquired or exacerbated in hospital, such as new infection, antimicrobial resistance, drug toxicity or malnutrition, which later results in death (Figure 1).

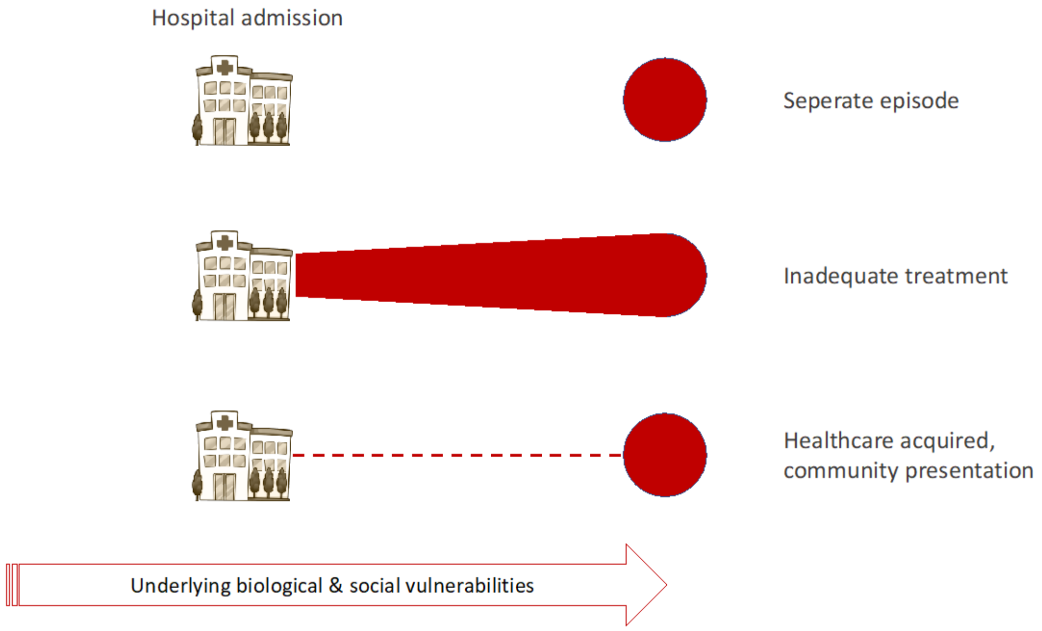

Figure 1. **Mechanisms of paediatric post-discharge mortality.**

Reference

1. The Childhood Acute Illness and Nutrition (CHAIN) Network. Childhood mortality during and after acute illness in Africa and south Asia: a prospective cohort study. Lancet Glob Health. 2022; 10: e673-e684.
